# Stability of consumer–resource interactions in periodic environments

**DOI:** 10.1098/rspb.2023.1636

**Published:** 2023-09-27

**Authors:** Carling Bieg, Gabriel Gellner, Kevin S. McCann

**Affiliations:** ^1^ Department of Integrative Biology, University of Guelph, Guelph, Ontario, Canada N1G 2W1; ^2^ Ecology and Evolutionary Biology, Yale University, New Haven, CT, USA

**Keywords:** consumer–resource interactions, chaos, oscillators, non-local dynamics, environmental periodicity, food web theory

## Abstract

Periodic fluctuations in abiotic conditions are ubiquitous across a range of temporal scales and regulate the structure and function of ecosystems through dynamic biotic responses that are adapted to these external forces. Research has suggested that certain environmental signatures may play a crucial role in the maintenance of biodiversity and the stability of food webs, while others argue that coupled oscillators ought to promote chaos. As such, numerous uncertainties remain regarding the intersection of temporal environmental patterns and biological responses, and we lack a general understanding of the implications for food web stability. Alarmingly, global change is altering the nature of both environmental rhythms and biological rates. Here, we develop a general theory for how continuous periodic variation in productivity, across temporal scales, influences the stability of consumer–resource interactions: a fundamental building block of food webs. Our results suggest that consumer–resource dynamics under environmental forcing are highly complex and depend on asymmetries in both the speed of forcing relative to underlying dynamics and in local stability properties. These asymmetries allow for environmentally driven stabilization under fast forcing, relative to underlying dynamics, as well as extremely complex and unstable dynamics at slower periodicities. Our results also suggest that changes in naturally occurring periodicities from climate change may lead to precipitous shifts in dynamics and stability.

## 1. Introduction

The natural environment is rife with noisy periodic signals, imperfect periodicities that are nonetheless regular. Depending on their longevity, organisms experience various environmental periodicities, from daily temperature swings to seasonal patterns in temperature and precipitation, to multi-year climatic patterns like the El-Niño Southern Oscillation (ENSO) [1,2]. Further, the regularity of these periodicities creates opportunities for organisms to adapt in anticipation of regularly changing environmental conditions (called ‘feedforward’ effects by Bernhardt *et al*. [3]), while simultaneously governed by intrinsic biotic periodicities that can also be mediated by the environment [4]. Given this, it is entirely possible that these fundamental periodicities could play a significant role in regulating the structure and function of ecosystems worldwide. Despite the recognition that such underlying periodicities abound and organisms have adapted to them, surprisingly little work has been done to incorporate these rhythms into our understanding of ecology [5]. This is more surprising considering climate change and, more broadly, global change are currently altering the nature of these underlying frequencies at a variety of scales [2].

While only modest amounts of work have focused on the intertwined abiotic and biotic rhythms that span ecological, spatial and temporal scales, and their impacts on ecosystem functioning from populations to whole food webs, researchers have characterized the structure of environmental variability [6]. Specifically, studies have repeatedly found that abiotic signals follow a 1/*f* scaling (*f* = frequency) suggesting that slow frequency periodicities (e.g. multi-decadal oscillations) drive a significant amount of environmental variation on top of the well-known rapid seasonal and diurnal periodicities [6,7]. Here, terrestrial systems show a more uniform distribution of frequencies (e.g. a more whitened noise) while aquatic and marine systems are skewed towards the lower frequencies characterizing brown and reddened noise [6,8]. As a first step towards bridging the gap between coupled environmental periodicities and ecosystem functioning, Mougi [5] used a spectrum of periodic environmental forcing to theoretically argue that species can coexist through trade-offs organized around the frequencies of nature's many rhythms, and Simon [9] showed that environmental frequencies may have complex interactions with cascading trophic dynamics. Further, researchers are beginning to show that some forms of periodic variation, like seasonality, may alter food web structure in a manner that maintains biodiversity by facilitating coexistence or stabilizing eco-evolutionary dynamics [10–12] and consumer–resource (C–R) interactions [13]. However, in many cases, these results depend on the interaction between extrinsic (environmental) and intrinsic (biotic) rhythms and the ability to adapt to changing environments. Thus, we are beginning to see some evidence that periodicities like seasonality may stabilize certain interactions, however, a general theoretical exploration into the potentially stabilizing mechanisms of environmental variation, across temporal scales, for food web dynamics is still in its infancy.

Despite some hints that seasonality may stabilize species interactions in some cases, theory has yet to make the link between the mathematical implications of adding another oscillator (i.e. periodic abiotic forcing is an oscillator) to C–R interactions and food web dynamics [14] and the broader, more biologically motivated explanations of C–R interactions in variable environments. As such, the literature is replete with mathematical analyses on the bifurcation structure of periodically forced models [15–17], which has emphasized the idea that the addition of an environmental oscillator to a biotic interaction often produces period-doubling cascades to chaos and complex dynamical phenomena. Consistent with these results, a separate more empirically motivated literature exists that argues for the increased presence of cycles in forced models [18–20]. However, what remains to be seen is how periodic environmental forcing interacts with underlying biotic rhythms in these systems. Furthermore, this theory has not explored these implications across the suite of abiotic and biotic rhythms that characterize natural systems, despite the nature of underlying dynamics (i.e. cycles) being clearly important in determining a system's response to environmental change and the combined timing of abiotic and biotic rhythms potentially determining a system's ultimate behaviour [21,22]. Altogether, research has yet to determine whether periodic forcing can be stabilizing relative to unforced food web interactions, despite the empirical or qualitative accounts of this [23], nor offered mechanistic insight into how forcing on different timescales alters C–R dynamics. We are therefore currently unequipped to tackle a mechanistic understanding of nature's vast rhythms.

**Figure 1.**
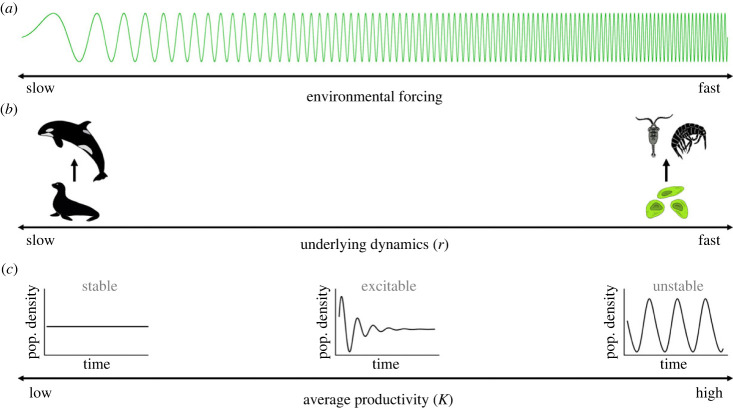
Model schematic and interactive factors regulating the speed and stability of C–R dynamics. We consider periodic environmental forcing across a range of periodicities (i.e. decomposing nature's signatures of variation) while also considering the speed of underlying dynamics, regulated by growth rates of C–R species pairs. Finally, we note that changing productivity (the environmental variable being forced temporally, *K*) alters the underlying stability of C–R interactions.

Here, we explore the role of extrinsic temporal forcing on C–R interactions, using graphical phaseplane analysis (among other techniques), to understand the nuances and implications of periodic environmental forcing for C–R dynamics and stability. We include forcing on various temporal scales (i.e. from daily to multi-decadal) by sinusoidally forcing primary productivity (*K*) around different mean values to reflect the fact that average productivity is increasing due to climate change. Finally, we use a range of growth rates to mimic varying life strategies (i.e. slow to fast pace of life) to test the generality of our results and suggest implications for future global change scenarios, as it is known that various anthropogenic stressors associated with global change (i.e. climate change, harvesting, land development, etc.) are simultaneously selecting for faster life strategies [24–26]. The speed of life strategies (i.e. growth rates) also dictates the speed of underlying dynamics in C–R systems (i.e. the periodicity of limit cycles), which we will show has an interactive effect with the speed of environmental forcing. Altogether, we explore the interactive effects of environmental forcing and C–R dynamics and thus the implications of changes in these fundamental fluctuations, across various temporal scales, the stability of whole food webs (figure 1).

## 2. Methods

### (a) Temporally forced consumer–resource model

We use an extension of the classic Rosenzweig–MacArthur C–R model [27] where we force resource carrying capacity (i.e. productivity), *K*, continuously to mimic fluctuations in productivity associated with environmental variability. Typically, fluctuations in temperature or precipitation (e.g. seasonality) will be reflected by similar changes in primary productivity. The Rosenzweig–MacArthur C–R model is an iconic model capable of reproducing equilibrium to cyclic dynamics that cover the range of dynamics found in nature [28], allowing us to explore the full range of dynamical possibilities with fluctuating *K*. *K* is known to drive transitions from monotonic approach to equilibrium (at low *K*), to excitable/overshoot and damped oscillations, and through a Hopf bifurcation (increasing *K*) leading to persistent oscillations (limit cycles) (figure 1).

We model this forcing in *K* as follows:

2.1 $$\begin{array}{rl} \frac{dC}{dt}= & C\left(\;\frac{eaR}{b+R}-d\right)\\ \mathrm{and}\;\frac{dR}{dt}= & R\left(\;r\left(1-\frac{R}{K_{\mathrm{mean}}+K_{\mathrm{force}}}\right)-\frac{aC}{b+R}\;\right), \end{array}\}$$

where *r* is the intrinsic rate of growth of the resource, *K*_mean_ is the average resource carrying capacity, *a* is consumer attack rate, *e* is the consumer's energy conversion efficiency, *b* is the half-saturation coefficient and *d* is consumer natural mortality.

Here, we force *K* sinusoidally as a function of time such that:

2.2 $$K_{\mathrm{force}}=A*\sin(\;p2\pi \;t),$$

where *A* is the amplitude of the *K* forcing (forcing occurs around *K*_mean_) and *p* is the forcing speed, such that the period (i.e. duration) of one *K* cycle is equal to *1/p*. It is worth noting that sinusoidal forcing is arguably the most simple way of incorporating environmental noise, but useful because it reflects highly autocorrelated environmental noise yet is continuously differentiable, allowing us to gain better analytical insight into the dynamics compared to discontinuous or random noise approaches.

We compare all simulation results to an unforced model (*A* = 0; *K*_force_ = 0). Numerical simulations were run in Wolfram Mathematica (v 12.3.1), and numerical results (i.e. coefficient of variation (CV) in C and R densities) were taken after a sufficiently long transient period to reach an asymptotic state (which ranged from 5000 to 90 000 time steps, depending on the forcing period). That is, since we vary the forcing speed (parameter *p*), the simulation length varied such that we could ensure our results captured sufficient information, while retaining computational efficiency. When calculating the CV of asymptotic behaviour, time series were simulated over at least 50 000 time steps, and the mean and standard deviation were sampled from a time span of either four full forcing periods (4/*p*) or 1000 time units, whichever was larger, at the end of each simulation. These CV values were compared to the CV of the deterministic system with *K* = *K*_mean_ to account for changes in CV and local stability with other model parameters (see electronic supplementary material, appendix figure A1).

### (b) Isoclines, equilibria and phaseplane analysis

While the isoclines for the Rozenzweig–MacArthur model have been studied extensively, we rely on these solutions and associated phaseplane analysis below, so we will review them briefly here. The isoclines for C and R in the classic (unforced) Rosenzweig–MacArthur model are found by setting each differential equation to 0 and solving for C or R for the purpose of visualizing our isoclines and model dynamics in the phaseplane later on.

2.3 $$\begin{array}{rl} \frac{dC}{dt}= & 0\;\mathrm{when}\;R=\frac{db}{ae-d}\\ \mathrm{and}\;\frac{dR}{dt}= & 0\;\mathrm{when}\;C=-\frac{r(b+R)(R-K)}{aK}. \end{array}\}$$

Here, we find the solution for the interior equilibrium (R*, C*) when

2.4 $$\frac{dC}{dt}=\frac{dR}{dt}=0,$$

that is, where the isoclines intersect:

2.5 $$\begin{array}{rl} R^{*}= & -\frac{bd}{d-ae}\\ \mathrm{and}\;C^{*}= & -\frac{be(bd+dK-aeK)r}{{\left(d-ae\right)}^{2}K}. \end{array}\}$$

### (c) Local stability analysis

We can find the symbolic function for the eigenvalues of the interior equilibrium by linearizing the model using the Jacobian matrix (with R* and C*), J:

2.6 $$J=\left[\begin{array}{cc} -\frac{dr(b(d+ae)+K(d-ae))}{\begin{array}{c} aeK(-d+ae) \end{array}} & \;\;-\frac{d}{e}\;\;\\ \frac{r(-bd-dK+aeK)}{aK} & \;\;\;\;\;0 \end{array}\right],$$

then taking the determinant of J, and simplifying using the characteristic equation. The dominant eigenvalues' (*λ_max_*) real part (ignoring the part of the characteristic equation under the square root sign, which is always negative, and therefore defines the imaginary part, when C–R dynamics are excitable and approaching the Hopf bifurcation) is equal to:

2.7 $$\lambda_{\max}=\frac{ader\;(-b\;+\;K)-d^{2}r\;(b\;+\;K)\;}{2aeK\;(-d\;+\;ae)}.$$

Notably, this solution is a function of *K*, with which we can use to symbolically determine where the Hopf bifurcation occurs (*λ*_max_(*K*) = 0) and how various parameters will influence this point. Clearly, other parameters can drive bifurcations (in fact, all parameters other than *r* have the potential to drive a Hopf bifurcation at some value > 0; for more information on this generality, see [29]), making this analysis and results potentially generalizable for periodic forcing of other rates, however, here we focus on the effect of changing productivity, *K*.

Thus, solving for *λ*_max_(*K*) = 0 gives us:

2.8 $$K_{\mathrm{Hopf}}=\frac{-b(d+ae)}{d-ae}.$$

We will use this symbolic solution for *K*_Hopf_ as a reference point for which to centre our numerical experiments around, therefore knowing the proportion of time in a cycle that is spent with a stable versus unstable equilibrium. For example, the point of symmetry where *K*_mean_ = *K*_Hopf_ is an interesting starting point as the system would spend equal time with an underlying deterministic skeleton being stable or unstable.

While environmental forcing ultimately creates a non-equilibrium problem, in situations where dynamics closely track a moving equilibrium (i.e. *K* influences equation (2.5)), local stability properties play an important role. We can use this symbolic solution for *λ*_max_(*K*) (equation (2.7)) to qualitatively understand characteristics of changing stability as the system fluctuates and passes through this important bifurcation point (e.g. *K*_Hopf_). Importantly, as *K* varies through time, the local stability (i.e. equation (2.7)) also changes, so we use a simple approximation as a first step towards understanding the role of local stability in our forced model. Towards understanding the influence of local stability as *K*, and therefore *λ*, fluctuates we can integrate *λ*_max_(*K*) over one full period of *K* forcing (i.e. equation (2.2); between *K*_min_ and *K*_max_), that is, from time = 0 to 1/*p*. This is an imperfect yet simple way of generalizing the attractive or repulsive ‘force’ near an equilibrium, as it perpetually changes in stability. Note that when *K*_mean_ = *K*_Hopf_, the eigenvalue is centred around 0 (i.e. *λ*_max_(*K*_Hopf_) = 0), however at different values of *K*_mean_ we correct for *λ* at *K*_mean_ (i.e. *λ*_max_(*K*_mean_)) to give us an estimate of the overall, or total, stability of our non-equilibrium model dynamics, relative to a deterministic model with static *K* at *K*_mean_.

In other words, our estimate for the standardized total local stability under non-equilibrium environmental forcing, relative to a constant environment, is:

2.9 $$\lambda_{\mathrm{total}}=\int_{0}^{1/p}\lambda_{\max}(K_{\mathrm{mean}}+K_{\mathrm{force}}(t))-\lambda_{\max}(K_{\mathrm{mean}})\;dt.$$

## 3. Results

### (a) Stability implications of forcing speed and underlying dynamics

**Figure 2.**
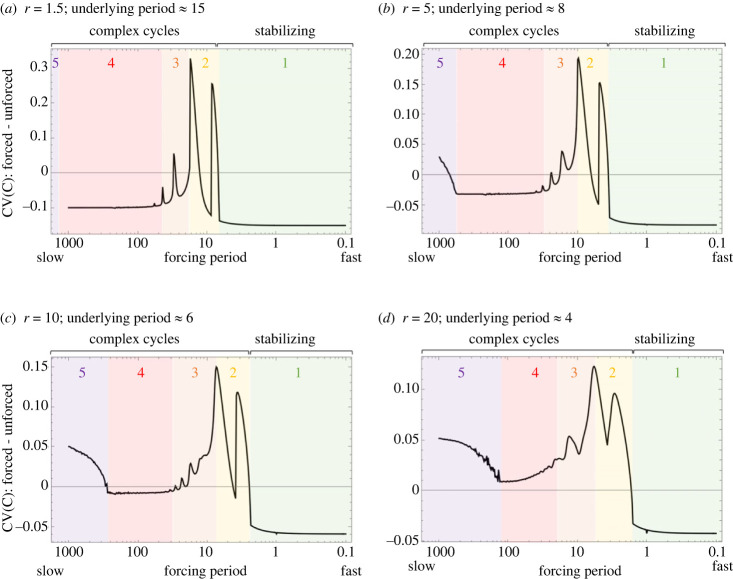
The effect of forcing period on stability (coefficient of variation, CV, of the consumer) of C–R dynamics in a forced system relative to an unforced model at the same mean *K* value. Panels (*a–d*) show this effect across different speeds of underlying dynamics (varying *r* values in panels (*a*) to (*d*): *r* = 1.5, 5, 10, 20 corresponds to approximate underlying periods for deterministic limit cycles (at *K*_mean_) of 15, 8, 6, 4 time units, respectively). Note coloured/numbered ‘zones’ 1–5 corresponding to forcing speeds with different dynamical implications, which we describe later on. Qualitatively we see a ‘stabilizing phase’ at fast environmental forcing speeds, and a ‘complex cycles phase,’ which depends on complex interactions between coupled oscillators, at slower frequencies. As a result of changing relative speeds of abiotic and biotic oscillators, the speed of underlying dynamics (i.e. resource growth rate, *r*) alters the magnitude of the stabilizing effect seen at fast frequencies, and shifts the transition between dynamical zones. Here, *K*_mean_ = *K*_Hopf_ + 0.05 (nearly symmetric forcing around Hopf), *A* = 0.5, *e* = 0.7, *a* = 1.3, *d* = 0.2, *b* = 1.

We find that the speed of forcing is highly influential on the resulting dynamical response of our C–R model and the stability of our forced (relative to unforced, see electronic supplementary material, figure A1) model with *K* = *K*_mean_ (figure 2). Figure 2 shows how the CV of the consumer, C, changes across forcing speeds (see electronic supplementary material, appendix figure A2 for R) when *K*_mean_ = *K*_Hopf_ + 0.05, that is, nearly symmetric forcing around the Hopf bifurcation point, or equal time spent with a stable versus unstable equilibrium (note *K* value slightly > *K*_Hopf_ chosen for visualization purposes to avoid degeneracy of deterministic model at *K* = *K*_Hopf_). Specifically, we see that fast periodic forcing can be stabilizing (CV forced – unforced < 0), however slow forcing has the potential to cause less stable, complex dynamics. While there are various forms of stability in ecological systems, we focus here on a common metric that easily unites theoretical and empirical approaches in ecology (that is, the coefficient of variation, CV). Notably, our results- are remarkably general across a range of *r* values (which dictates the speed of underlying C–R cycles [4], as shown in electronic supplementary material, figure A1 and noted in figure 2), as well as other model parameters, including average *K* (which dictates underlying stability and energy flux; electronic supplementary material, appendix figure A3) and *e* (which regulates the numerical response in C; electronic supplementary material, appendix figure A4). Note that while there are some differences in the degree to which periodic forcing is stabilizing or destabilizing in figure 2*a–d*, the general pattern, despite its nonlinearity, remains consistent. Along these lines, we note that the implications for stability are dependent on the *relative* speeds of oscillators (forcing and underlying dynamics) such that faster system dynamics (high *r*) will become destabilized at slightly faster forcing frequencies (*p*) than an overall slower system (lower *r*). In other words, while this result is general across different biological rates, increasing growth rates (*r*) seem to decrease the potential for environmental forcing to drive stabilization. Due to this generality, we highlight here that there are clearly distinct dynamical regions or ‘zones’ (figure 2) that characterize different interactions between the coupled oscillators (i.e. excitable C–R interaction and our environmental forcing).

To mechanistically explore the role of periodic forcing on the stability of C–R interactions, we first separate these enumerated and colour-coded zones from figure 2 into two distinct phases that we will refer to as the ‘*stabilizing phase*’ (fast periodic forcing relative to underlying dynamics; green zone 1) and ‘*complex cycles phase*’ (relatively slow forcing; yellow to purple zone 2–5) (figure 2), and then qualitatively explore each of these five zones separately. We note the interesting nonlinear trend in stability seen in figure 2, such that we see a ‘tongue of destabilization’ at moderate–slow forcing speeds, which suggests a complex interaction between oscillators that we will explore in more detail below. Furthermore, we refer to these two distinct regions despite there clearly being multiple dynamical zones due to an interesting interplay between local and nonlocal dynamics.

### (b) Asymmetry in local stability: stabilizing phase

First, we explore the role of high frequency periodic environmental forcing on C–R dynamics, that is, the ‘*stabilizing phase*’ in figure 2 (green zone 1). Here, we see that fast forcing stabilizes dynamics relative to an unforced model at the same average *K* value. Since increasing average productivity (*K*) destabilizes C–R dynamics in general (figure 1; also see box 1), this result seems to initially suggest that our system might be responding to a lower ‘realized’ *K* value (e.g. compared to the arithmetic mean of *K*) over the range of forcing, a result that would be akin to Jensen's inequality for variable environments [31]. Indeed, previous research has shown that periodic forcing of *K* in the logistic equation (i.e. no C) results in lower mean densities, effectively lowering the ‘realized’ *K* [32], but the addition of C makes the outcomes of *K* forcing less intuitive. That is, it is nonlinearities in the dynamical responses of both R and C that ought to be driving these results.

Box 1. **Background on C–R phaseplane.**

Before discussing these dynamical responses in more detail using the phaseplane, we will discuss some important background information regarding the geometry (i.e. phaseplane) of the Rosenzweig–MacArthur C–R model [27] and on the effects of changing *K* in the deterministic skeleton (i.e. no forcing).

Figure B1*a* shows how changing *K* qualitatively alters the phaseplane geometry. First, and importantly, note that changing *K* alters the equilibrium coordinates and stability of that equilibrium. Specifically, the C isocline is not affected by changing *K*, but the R isocline is ‘stretched’ as *K* increases the C = 0 intercept (R = *K* is an axial equilibrium). This ultimately changes the location of the interior equilibrium (C and R coordinates, where isoclines intersect) solely in the ‘vertical’ (C) direction (figure B1*a*; also see equation (2.4)). So, as *K* fluctuates, the interior equilibrium moves in the phaseplane in the C direction as it also fluctuates in stability. 
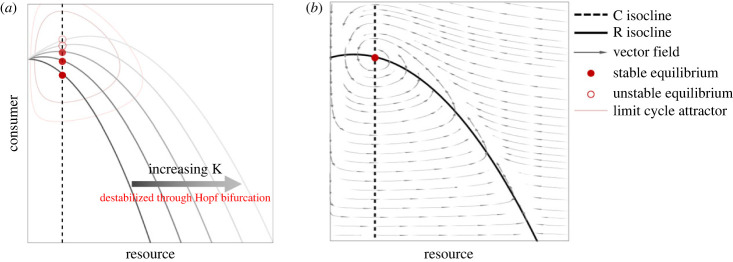

**Figure B1.** Graphical representation of changing resource carrying capacity (i.e. productivity, *K*) in a Rosenzweig–MacArthur consumer (C)–resource (R) model. (*a*) Showing how the isocline geometry and equilibrium structure changes with increasing *K*; and (*b*) demonstrating the underlying vector field of the phaseplane around a stable (but excitable) equilibrium.

Second, and as previously mentioned, it is also important to note that increasing *K* increases the excitability (overshoot potential; complex eigenvalues) of C–R dynamics, until eventually driving a Hopf bifurcation (transition from a stable equilibrium to limit cycles, figure B1*a*; figure 1). The changing attractor (equilibrium or limit cycle) displayed in figure B1*a* is a useful reference point for the dynamical response of forcing *K*. Note that this general pattern is interestingly what explains the paradox of enrichment [30], and is in fact qualitatively consistent for any parameter that increases the strength of coupling between C and R (i.e. increasing *a*, *e*, *b* or decreasing *d* in our model [29]). Therefore, as *K* is forced periodically, the underlying excitability (and stability if being forced past the Hopf bifurcation point) is fundamentally and perpetually changing over time.

Finally, it is worth noting that C–R models are typically ‘fast’ in the R direction such that in a deterministic scenario the system will respond quickly in R, reach the R isocline (or potentially overshoot), and then more slowly approach the equilibrium (i.e. follow vector field in figure B1*b*). This of course depends on the relative speeds of C and R, but given resources are generally faster than consumers, this is a fairly robust pattern. Also of note is that when dynamics are excitable but the equilibrium is still stable (i.e. phaseplane shown in figure B1*b*), a perturbation in a deterministic system would result in damped cycles as it returns to the equilibrium (i.e. not monotonic).

This phaseplane description becomes important when evaluating a system's response to a perturbation off the equilibrium, and whether the system will respond monotonically back to equilibrium or ‘cycle’ around as it returns (or away if unstable, in our case towards a limit cycle attractor). This also of course determines how a system may track a moving equilibrium. Effectively, a moving equilibrium is similar to a perturbation off a stationary equilibrium, with the exception that in our case the equilibrium is perpetually moving in the phaseplane.

**Figure 3.**
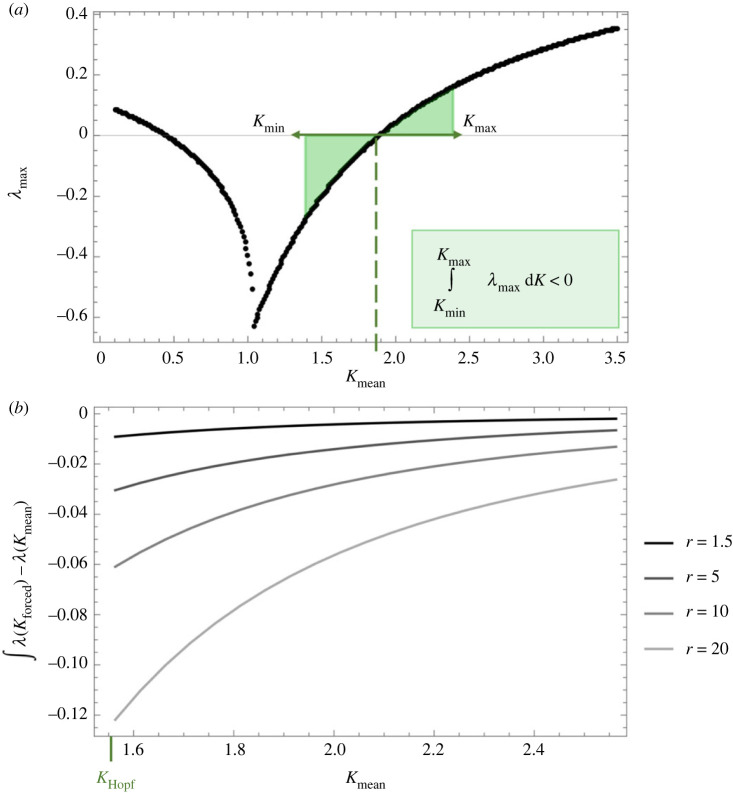
Unpacking the stabilizing phase (fast environmental periodicities). (*a*) Asymmetry in local stability explains the stabilizing effect at fast forcing speeds. That is, the integral of the maximum eigenvalue over the range of *K* forcing is always negative. (*b*) Despite always being negative, the integral is dependent on other model parameters (notably *r*) as they alter the shape of the curve displayed in (*a*). Note that while the change in the eigenvalue integral across *K*_mean_ remains consistent with changing *r*, the magnitude of the asymmetry significantly varies. Here, *A* = 0.5, *e* = 0.7, *a* = 1.3, *d* = 0.2, *b* = 1, *p* = 1.

Interestingly, this nonlinear dynamical response can begin to be explored through local stability analysis. Since the system's dynamics do not seem to travel far from the equilibrium, even though the equilibrium coordinates as well as its local stability (i.e. *λ*_max_) are changing with our fluctuating *K* (e.g. equation(s) (2.5) and (2.7)), local stability properties may explain a surprising amount of our stabilizing effect under fast environmental forcing. To capture a measure of the overall stability of our non-equilibrium (forced) model (i.e. the net attracting or repulsing force to or from an equilibrium), we take the integral of *λ*_max_ (real part of the maximum eigenvalue, equation (2.7), hereafter referred to simply as *λ*) over a full period of *K* forcing (i.e. between *K*_min_ and *K*_max_) and standardize for *λ* at *K*_mean_ (see Methods). We refer to this integral as *λ*_total_ (equation (2.9)). We find that *λ*_total_ is always negative, as long as *K*_min_ is greater than the bottom of the eigenvalue ‘checkmark’ in figure 3*a* (most stable point), where *λ* becomes complex and the C–R dynamics become excitable (note, though, that below the Hopf bifurcation point, where *λ* = 0, the deterministic system is always stable so here we focus only on more interesting areas of parameter space above this point). This result suggests a consistently stabilizing effect of forcing *K* due to asymmetry in local stability (figure 3*a*). This negative integral indicates that locally, the dynamics are more strongly influenced by stabilizing than destabilizing forces due to an asymmetry in local stability properties over a range in *K* values. Note that this general result is consistent across various parameters, however the magnitude of this asymmetry may be influenced by other factors such as the speed, or responsiveness, of the underlying system. That is, resource growth rate, *r*, increases this asymmetry in local stability, particularly around the Hopf bifurcation point (figure 3*b*) as it changes the slope of the eigenvalue curve (electronic supplementary material, figure A1). Furthermore, we should note that while we use the Rosenzweig–MacArthur C–R model here, which undergoes a Hopf bifurcation, the general convex nature of this eigenvalue curve (figure 3*a*) is ubiquitous across C–R systems regardless of the Hopf bifurcation potential (e.g. Lotka–Volterra with logistic growth, C–R models with Type III functional responses, and those with consumer self-limitation; [29,33,34]). In other words, this negative eigenvalue integral, centred around the mean, result ought to generally be true for all C–R systems.

To summarize, fast environmental forcing can have a stabilizing effect driven by asymmetries in local stability. However, as the speed of our forcing slows, the system can travel further away from the equilibrium at *K*_mean_, or has more time to do so, and is thus less influenced by local stability as it picks up non-local dynamics.

### (c) Nature of the dynamical response to forcing: complex cycles phase

As periodic environmental forcing slows, we see complex, non-local dynamics. Here, we explore the role of forcing in this ‘*complex cycles phase*’ shown in figure 2 (zones 2–5). At this point, local dynamics are seemingly overwhelmed by non-local dynamics due to the slowing periodic forcing driving an abrupt shift in the resulting oscillator and stability relative to the underlying deterministic system (i.e. between zones 1 and 2, figure 2). As such, we here focus on the nature of non-local dynamics to explore our complex cycles phase. In this exploration we will rely heavily on the phaseplane, and have provided background information on the C–R phaseplane in box 1 (also see Methods for isocline solutions). We do so due to the usefulness of using underlying model geometry (i.e. isoclines) as reference points for understanding the system dynamics and thus the generality of the different dynamical zones shown in figure 2. Phaseplanes have been used for understanding dynamics since the initial introduction to the Rosenzweig–MacArthur model [27], and thus we keep with this tradition as way to understand the general nature of attractors and move from characterizing local to global dynamics[29].

**Figure 4.**
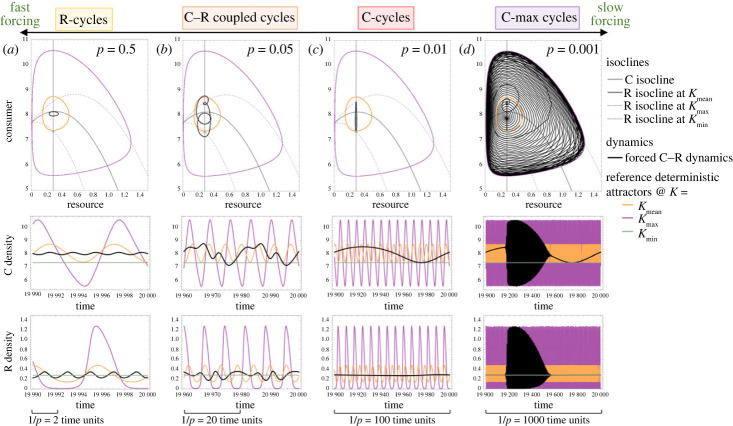
Dynamical characteristics of complex cycles phase with changing environmental forcing speeds (*p*). Dynamics in each zone result from asymmetry in relative timescales of coupled oscillators, and display routes to chaos with changing forcing speed. Dynamics shown here in the phaseplane and as corresponding time series are representative of each zone highlighted in figure 2 (colour-coded and enumerated; panels (*a*–*d*) reflect zones 2–5). Here, *K*_mean_ = *K*_Hopf_ + 0.05 (i.e. nearly symmetric forcing around *K*_Hopf_ but such that the deterministic system at *K*_mean_ displays stable limit cycles). Also shown are reference attractors for underlying deterministic models as coloured lines representing *K*_min_, *K*_mean_ and *K*_max_ in green, orange and purple, respectively. In all cases, *A* = 0.5, *r* = 10, *e* = 0.7, *a* = 1.3, *d* = 0.2, *b* = 1.

With changing forcing speeds, we see the system go through a consistent transition through qualitatively different dynamical responses as the speed of forcing slows, and here we explore this transition in the phaseplane and with corresponding time series, such that we can compare our forced system's dynamics to various attractors (i.e. relative to attractors, that is, the attracting stable equilibrium or limit cycles, at *K*_min_, *K*_mean_ and *K*_max_ as benchmarks from deterministic models; figure 4). Specifically, with slowing forcing speed we refer to the stages of this transition (i.e. zones 2–5; figure 2) as R-cycles (yellow, zone 2), C–R cycles (orange, zone 3), C-cycles (red, zone 4), and finally C–R max/boundary cycles (purple, zone 5) (figure 4). Here, we briefly discuss each of these ‘stages.’ While the dynamical response to changing forcing speed is truly a continuous and complex transition, characterized by responses in both C and R and complex interactions between coupled oscillators, the apparent discontinuity seen in figure 2 motivates us to explore each of these qualitative zones separately. Thus, this categorical explanation of these ‘stages’, or routes to chaos with the slowing of environmental forcing, can help us begin to explore the interaction between multiple oscillators at various timescales and the asymmetries within them.

Initially, our model dynamics are largely responsive in the R-direction of the phaseplane; thus we denote these as ‘R-cycles’ (figure 4*a*). To conceptualize the dynamical responses, we can think about these results in terms of one ‘cycle’ and the system's response: since R is relatively faster than C (see box 1) we see a response in R to changing *K* (note it technically gets ‘knocked off’ the equilibrium since the equilibrium itself is changing), similar to overshoot dynamics, but there is not enough time for C to respond before *K* once again returns. This result is similar to what we see in the ‘stabilizing zone’ discussed above, but there is enough time to allow for a small dynamical response in R to the changing *K* (i.e. larger amplitude of R-cycles as forcing is slightly slowed). This also leads to the next phase of our dynamical transition with decreasing forcing speed. That is, what we refer to as ‘C–R cycles’ (figure 4*b*). Here, the forcing is effectively slow enough to cause responses in both C and R, which results in much more complex dynamics. In this case, the relative speeds of environmental forcing and the underlying dynamics are more equal in magnitude, which manifests in more typical coupled oscillators as the system tries to keep up with the constantly moving equilibrium while also contending with its own excitability and non-local dynamics. Notably, coupled oscillators are a recipe for chaos [35], and while we do not see true chaos here, since the asymptotic behaviour resembles consistent and repeatable behaviour, this phase of coupled oscillators explains our ‘tongue of destabilization’ shown in figure 2.

Next, with further slowing of environmental forcing (increasing periodicity), we find an interesting transition to what we refer to here as ‘C-cycles’ (figure 4*c*). In this case, the dynamics begin to closely track the slowly moving equilibrium (moving in the C-direction as noted above, box 1). Here, the equilibrium shifts slowly such that we do not see the same overshoot as in the initial R-cycles; the dynamics are equilibrium dominated in a slightly different way than described in our previous section, but we note the relative decrease in CV during this phase (figure 2). That is, the dynamics are practically invariant in R; once ‘locked on’ to the equilibrium, R does not change. While environmental forcing is slow enough to allow for this equilibrium tracking, the system is unable to adopt the unstable limit cycles at *K_max_* (or anywhere > *K*_Hopf_) before *K* again retreats. Finally, with extremely slow forcing, we characterize the response as ‘C–R max/boundary cycles' (figure 4*d*), as the system perfectly tracks the changing equilibrium (or limit cycle boundary once past *K*_Hopf_) with slowly changing *K*. This final dynamical response is similar to what are known as relaxation oscillators [36,37] and bursting oscillations [38] in the applied mathematical literature.

In short, the complex dynamical response to environmental forcing is dependent on asymmetries between the speeds of environmental forcing and underlying dynamics, as well as asymmetries in the relative speeds and individual responsiveness of R and C. As such, the transition between phases we describe here may occur across different ranges of forcing speeds with different underlying dynamics (e.g. lower *r*), as also suggested in figure 2, however the qualitative phases of this response are consistent (see electronic supplementary material, appendix figure A5). In other words, the speed of underlying dynamics will determine how a C–R interaction responds to different periodicities. Note also that the transition between dynamical phases can also be inferred through bifurcation diagrams (electronic supplementary material, appendix figure A6) such that we see clear cascades of increasing cycle complexity and reversal, i.e. the route to chaos, with changing forcing speeds. This could have drastic implications for both changing environmental signals and changing life histories, particularly if a system is near a boundary between our dynamical zones.

## 4. Discussion

We have shown here that the implications of environmental periodicities for food web stability are highly complex and depend on multiple asymmetries: asymmetries in the speed of forcing relative to underlying dynamics (figure 2), asymmetries in local stability properties (figure 3), and asymmetries in the speeds of R and C (i.e. growth rates and energy flux; box 1, figure 4). Altogether, these asymmetries interact to determine the dynamical response to environmental forcing and implications for stability of C–R interactions. Note that when the system is always within stable parameter space (i.e. an unforced model over the full range of *K* is stable, that is, <*K*_Hopf_), the forcing is clearly destabilizing by adding oscillatory dynamics to otherwise invariant populations, but the extent of this is dependent on things like biological rates (i.e. *r*) and forcing speed, with the same general dynamical pattern remaining consistent. That is, slow forcing is always more destabilizing than fast forcing, and we consistently see this general transition between dynamical responses (i.e. the nonlinear pattern first shown in figure 2).

Importantly, the effect of forcing depends on an interesting relationship between local stability (stabilizing) and non-local dynamics (complex and destabilizing). Specifically, we found that fast forcing, relative to the speed of underlying dynamics, can stabilize excitable dynamics, but the reverse is also true, slow forcing is destabilizing (i.e. figure 2). Additionally, under certain intermediate conditions, temporal forcing can interact with the underlying dynamics in complex ways through the effect of coupling these oscillators and driving increased instability; this happens when the forcing and underlying dynamics have similar influence (influence on dynamics is related to relative speeds) or periodicities (e.g. figure 4), similar to results regarding the amplification of noise when periodicities are aligned in other models [22,39,40].

As a first step towards describing this complex and interactive effect of coupled oscillators (abiotic and biotic rhythms), we characterized the effects of environmental forcing across a range of temporal scales by describing qualitatively consistent ‘zones’ of dynamical responses, based on the dynamic responses of R and C, as dynamics transition towards chaos with decreasing forcing speeds (figures 2 and 4). Notably, we emphasize the generality of our results and the interactive effects of environmental forcing. Our work is the first that we are aware of that has characterized C–R dynamics, across timescales of periodic forcing, in a generalizable way. It is important to note that our approach was intentionally general as this adds strength to understanding these broad patterns. In fact, our phaseplane approach to interpreting these complex cycles may have the potential to aid generalizable understanding of the role of periodic variation in other parameters that have qualitatively similar effects on underlying stability [29] (e.g. periodic foraging behaviour or response to the environment via *a*, or environmentally variable metabolic costs via *d*). However, it simultaneously urges us to harness the empirical literature to determine where we are (e.g. biological rates scaled to environmental rhythms) and where we may be going (i.e. under various global changes). Our results suggest that slight changes to biological rates or the environment may have drastic consequences to the dynamics and stability of food webs, especially if systems are currently near the boundaries between our dynamical ‘zones.’ The discontinuity between these zones suggests that slight environmental or biotic shifts could have abrupt and drastic effects on the stability of C–R interactions and ultimately food webs.

**Figure 5.**
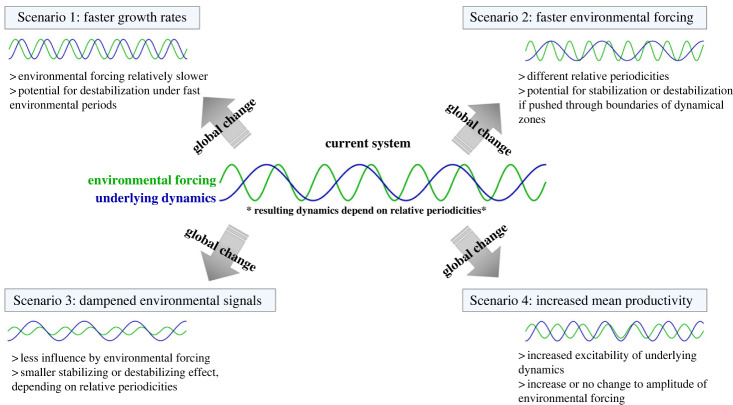
Scenarios of global change and implications for abiotic (environmental) and biotic periodicities. The implications for stability depend on initial conditions for both environmental rhythms and underlying food web dynamics, but potential outcomes of these changes are described based on our theoretical results.

Thus, our results have clear implications for global change, and highlight the importance of developing a theoretical understanding of the effects of environmental variability and integrating this with the empirical literature (figure 5). While a mechanistic understanding of these complex dynamics is still in its infancy, it is alarming that both biological rates and the nature of environmental fluctuations appear to be fundamentally changing in the face of climate change and other anthropogenic impacts. For example, in addition to increases in average primary production due to climate change and anthropogenic land use (e.g. nutrient run-off), human impacts (including warming) are driving widespread selection for fast life strategies and associated traits (i.e. smaller body sizes and faster growth rates within and among populations; [24,25]). At the same time, some of the ‘fast’ environmental periodicities are becoming dampened: nights and winters are becoming both shorter and warmer [2,41–43], and wet/dry seasonal patterns in the tropics are being altered due to a combination of climate change and hydrological development [44]. It is less clear if the periodicities of longer-term fluctuations are changing (i.e. versus the periodicities becoming more noisy), but evidence is beginning to suggest that multi-year cycles like ENSO are increasing in frequency [2,45]. Regardless, it is clear the environment is fundamentally changing, and the implications for ecological functioning could be drastic.

The simultaneous effects of speeding up biological rates and altered environmental signals may have severe consequences for the stability and maintenance of biodiversity, with the potential for highly complex coupled oscillators, including chaos, in what were formerly environmentally stabilized systems (figure 5). While the implications of global change require further empirical work to determine what dynamical regime particular systems currently reside in, we outline several potential global change scenarios in figure 5 based on changes to environmental or biotic periodicities. Due to the interaction between these changing periodicities, it is entirely possible that global changes may push systems between qualitative dynamical regimes, having potentially catastrophic implications for ecosystem structure and function. Furthermore, increased ‘reddening’ of environmental noise (increased autocorrelation of environmental patterns) with climate change could be introducing altogether new periodicities with unknown implications for food web stability [46]. Our results show that depending on the frequency of these newly introduced cycles, there is the potential for highly complex dynamics and chaos, particularly when coupled with previously occurring oscillators.

Here, we have explored the effect of periodic forcing, across temporal scales, in simple C–R interactions and our results imply that global change is likely to alter the nature of dynamics throughout whole food webs. As these complex oscillators interact and cascade through the ecological hierarchy, the implications for stability of whole food webs remains uncertain. Ultimately, integrating the nature of true environmental patterning (i.e. multiple abiotic and biotic rhythms) with a theory for whole food webs is needed for a deeper understanding of ecosystem functioning in variable environments. The dynamic and complex results seen here give us a glimpse into a future world of widespread biotic and abiotic change and the interactive nature of these rhythms.

## Data Availability

Mathematica code used for analysis is available on GitHub: https://github.com/carlingbieg/CR_Kforcing or Zenodo https://doi.org/10.5281/zenodo.8263034 [47]. The data are provided in the electronic supplementary material [48].

## References

[1] von der Heydt AS, Ashwin P, Camp CD, Crucifix M, Dijkstra HA, Ditlevsen P, Lenton TM. 2021 Quantification and interpretation of the climate variability record. Glob. Planet. Change **197**, 103399. (10.1016/j.gloplacha.2020.103399)

[2] Dillon ME, Woods HA, Wang G, Fey SB, Vasseur DA, Telemeco RS, Marshall K, Pincebourde S. 2016 Life in the frequency domain: the biological impacts of changes in climate variability at multiple time scales. Integr. Comp. Biol. **56**, 14-30. (10.1093/icb/icw024) 27252201

[3] Bernhardt JR, O'Connor MI, Sunday JM, Gonzalez A. 2020 Life in fluctuating environments. Phil. Trans. R. Soc. B **375**, 20190454. (10.1098/rstb.2019.0454) 33131443 PMC7662201

[4] Rubin JE, Earn DJD, Greenwood PE, Parsons TL, Abbott KC. 2022 Irregular population cycles driven by environmental stochasticity and saddle crawlbys. Oikos **2023**, e09290. (10.1111/oik.09290)

[5] Mougi A. 2020 Polyrhythmic foraging and competitive coexistence. Sci. Rep. **10**, 20282. (10.1038/s41598-020-77483-3) 33219304 PMC7679447

[6] Vasseur DA, Yodzis P. 2004 The color of environmental noise. Ecology **85**, 1146-1152. (10.1890/02-3122)

[7] Halley JM. 1996 Ecology, evolution and 1f-noise. Trends Ecol. Evol. **11**, 33-37. (10.1016/0169-5347(96)81067-6) 21237757

[8] Steele JH. 1985 A comparison of terrestrial and marine ecological systems. Nature **313**, 355-358. (10.1038/313355A0)

[9] Simon FW, Vasseur DA. 2021 Variation cascades: resource pulses and top-down effects across time scales. Ecology **102**, e03277. (10.1002/ECY.3277) 33354775

[10] McMeans BC, McCann KS, Humphries M, Rooney N, Fisk AT. 2015 Food web structure in temporally-forced ecosystems. Trends Ecol. Evol. **30**, 662-672. (10.1016/J.TREE.2015.09.001) 26452520

[11] Takimoto G, Iwata T, Murakami M. 2002 Seasonal subsidy stabilizes food web dynamics: balance in a heterogeneous landscape. Ecol. Res. **17**, 433-439. (10.1046/j.1440-1703.2002.00502.x)

[12] Miller ET, Klausmeier CA. 2017 Evolutionary stability of coexistence due to the storage effect in a two-season model. Theor. Ecol. **10**, 91-103. (10.1007/s12080-016-0314-z)

[13] Twardochleb LA, Zarnetske PL, Klausmeier CA. 2023 Life-history responses to temperature and seasonality mediate ectotherm consumer–resource dynamics under climate warming. Proc. R. Soc. B **290**, 20222377. (10.1098/rspb.2022.2377) PMC10130723 37122251

[14] White ER, Hastings A. 2020 Seasonality in ecology: progress and prospects in theory. Ecol. Complex. **44**, 100867. (10.1016/j.ecocom.2020.100867)

[15] Gragnani A, Rinaldi S. 1995 A universal bifurcation diagram for seasonally perturbed predator–prey models. Bull. Math. Biol. **57**, 701-712. (10.1007/BF02461847)

[16] Ma J, Xu Y, Xu W, Li Y, Kurths J. 2019 Slowing down critical transitions via Gaussian white noise and periodic force. Sci. China Tech. Sci. **62**, 2144-2152. (10.1007/s11431-019-9557-2)

[17] Kuznetsov YA, Muratori S, Rinaldi S. 1992 Bifurcations and chaos in a periodic predator–prey model. Int. J. Bifurc. Chaos **02**, 117-128. (10.1142/S0218127492000112)

[18] Hanski I, Korpimaki E. 1995 Microtine rodent dynamics in Northern Europe: parameterized models for the predator–prey interaction. Ecology **76**, 840-850.

[19] Taylor RA, White A, Sherratt JA. 2013 How do variations in seasonality affect population cycles? Proc. R. Soc. B Biol. Sci. **280**, 20122714. (10.1098/rspb.2012.2714) PMC3574328 23325773

[20] Taylor RA, Sherratt JA, White A. 2013 Seasonal forcing and multi-year cycles in interacting populations: lessons from a predator–prey model. J. Math. Biol. **67**, 1741-1764. (10.1007/s00285-012-0612-z) 23138231

[21] Alkhayuon H, Tyson RC, Wieczorek S. 2021 Phase tipping: how cyclic ecosystems respond to contemporary climate. Proc. R. Soc. A Math. Phys. Eng. Sci. **477**, 20210059. (10.1098/rspa.2021.0059) PMC8511773 35153584

[22] Greenman JV, Benton TG. 2003 The amplification of environmental noise in population models: causes and consequences. Am. Nat. **161**, 225-239. (10.1086/345784) 12675369

[23] Junk WJ, Bayley PB, Sparks RE. 1989 The flood pulse concept in river-floodplain systems. Can. J. Fish. Aquat. Sci. Spec. Publ. **106**, 110-127. (https://www.researchgate.net/publication/256981220_The_Flood_Pulse_Concept_in_River-Floodplain_Systems)

[24] Wang H-Y, Shen S-F, Chen Y-S, Kiang Y-K, Heino M. 2020 Life histories determine divergent population trends for fishes under climate warming. Nat. Commun. **11**, 4088. (10.1038/s41467-020-17937-4) 32796849 PMC7428017

[25] Gardner JL, Peters A, Kearney MR, Joseph L, Heinsohn R. 2011 Declining body size: a third universal response to warming? Trends Ecol. Evol. **26**, 285-291. (10.1016/j.tree.2011.03.005) 21470708

[26] Johnston EL, Clark GF, Bruno JF. 2022 The speeding up of marine ecosystems. Clim. Chang. Ecol. **3**, 100055. (10.1016/j.ecochg.2022.100055)

[27] Rosenzweig ML, MacArthur RH. 1963 Graphical representation and stability conditions of predator–prey interactions. Am. Nat. **97**, 209-223.

[28] Kendall BE, Briggs CJ, Murdoch WW, Turchin P, Ellner SP, McCauley E, Nisbet RM, Wood SN. 1999 Why do populations cycle? A synthesis of statistical and mechanistic modeling approaches. Ecology **80**, 1789-1805. (10.1890/0012-9658(1999)080[1789:WDPCAS]2.0.CO;2)

[29] McCann KS. 2011 Food webs (MPB-50). Princeton, NJ: Princeton University Press.

[30] Rosenzweig ML. 1971 Paradox of enrichment: destabilization of exploitation ecosystems in ecological time. Science **171**, 385-387. (10.1126/science.171.3969.385) 5538935

[31] Ruel JJ, Ayres MP. 1999 Jensen's inequality predicts effects of environmental variation. Trends Ecol. Evol. **14**, 361-366. 10441312 10.1016/s0169-5347(99)01664-x

[32] Rosenblat S. 1980 Population models in a periodically fluctuating environment. J. Math. Biol. **9**, 23-36. (10.1007/BF00276033)

[33] Gellner G, McCann KS. 2016 Consistent role of weak and strong interactions in high- and low-diversity trophic food webs. Nat. Commun. **7**, 11180. (10.1038/ncomms11180) 27068000 PMC4832055

[34] Gellner G, Mccann KS, Hastings A. 2016 The duality of stability: towards a stochastic theory of species interactions. Theor. Ecol. **9**, 477-485. (10.1007/s12080-016-0303-2)

[35] MacDonald N. 1978 Coupled oscillators in chaotic modes. Nature **274**, 847. (10.1038/274847a0)

[36] Kolesov AI, Kolesov AI, Kolesov AY, Kolesov YS, Kolesov IS. 1995 Relaxation oscillations in mathematical models of ecology. Providence, RI: American Mathematical Society.

[37] Kolesov AY, Rozov NK. 2011 The theory of relaxation oscillations for Hutchinson's equation. Sb. Math. **202**, 829-858. (10.1070/SM2011v202n06ABEH004168)

[38] Xindong M, Shuqian C, Hulun G. 2017 Routes to bursting oscillations in a modified van der Pol–Duffing oscillator with slow-varying periodic excitation. J. Vib. Control, 107754631774002. (10.1177/1077546317740020)

[39] Greenman JV, Benton TG. 2005 The frequency spectrum of structured discrete time population models: its properties and their ecological implications. Oikos **110**, 369-389. (10.1111/j.0030-1299.2005.13652.x)

[40] Touzot L, Venner S, Baubet É, Rousset C, Gaillard JM, Gamelon M. 2023 Amplified cyclicality in mast seeding dynamics positively influences the dynamics of a seed consumer species. Am. Nat. **201**, 38-51. (10.1086/721905) 36524926

[41] Woolway RI et al. 2021 Phenological shifts in lake stratification under climate change. Nat. Commun. **12**, 2318. (10.1038/s41467-021-22657-4) 33875656 PMC8055693

[42] Sharma S et al. 2019 Widespread loss of lake ice around the Northern Hemisphere in a warming world. Nat. Clim. Chang. **9**, 227-231. (10.1038/s41558-018-0393-5)

[43] Sparks TH, Menzel A. 2002 Observed changes in seasons: an overview. Int. J. Climatol. **22**, 1715-1725. (10.1002/joc.821)

[44] Arias ME, Cochrane TA, Piman T, Kummu M, Caruso BS, Killeen TJ. 2012 Quantifying changes in flooding and habitats in the Tonle Sap Lake (Cambodia) caused by water infrastructure development and climate change in the Mekong Basin. J. Environ. Manage. **112**, 53-66. (10.1016/j.jenvman.2012.07.003) 22877742

[45] Yeh SW, Kug JS, Dewitte B, Kwon MH, Kirtman BP, Jin FF. 2009 El-Niño in a changing climate. Nature **461**, 511-514. (10.1038/nature08316) 19779449

[46] Van Der Bolt B, Van Nes EH, Bathiany S, Vollebregt ME, Scheffer M. 2018 Climate reddening increases the chance of critical transitions. Nat. Clim. Chang. **8**, 478-484. (10.1038/s41558-018-0160-7)

[47] Bieg C. 2023 Code for: Stability of consumer–resource interactions in periodic environments. Zenodo. (10.5281/zenodo.8263034) PMC10523078 37752846

[48] Bieg C, Gellner G, McCann KS. 2023 Stability of consumer–resource interactions in periodic environments. Figshare. (10.6084/m9.figshare.c.6836726) PMC10523078 37752846

